# Incidence of childhood overweight and obesity and its association with weight-related attitudes and behaviors in China: a national longitudinal study

**DOI:** 10.1186/s12966-018-0737-6

**Published:** 2018-11-03

**Authors:** Li Cai, Meixia Dai, Lizi Lin, Wenhan Yang, Yajun Chen, Jun Ma, Jin Jing

**Affiliations:** 1 Department of Maternal and Child Health, School of Public Health, Sun Yat-Sen University, Guangzhou, China; 2 School of Public Health, Peking University, Beijing, China

**Keywords:** Childhood obesity, Incidence, Weight-change intention, Weight-control practices, Behavioral change

## Abstract

**Background:**

Childhood obesity is a major public health challenge. We aimed to investigate the incidence of overweight/obesity among Chinese children; and prospectively examine the associations of weight-change intention with risk of overweight/obesity and behavioral changes in initially normal-weight children.

**Methods:**

A national sample of 21,796 children aged 6–17 years were investigated in September 2013 and followed up nine months later, of which 19,887 (91%) were normal weight and 1909 (9%) were underweight at baseline. Weight and height were objectively measured. Weight perception, weight-change intention, weight control practices, weight-related behaviors, and demographic information were obtained by questionnaires.

**Results:**

Among children with underweight or normal-weight, the cumulative incidence of overweight/obesity was 2.77% (95% CI 2.55–2.99). Among normal-weight children, those who overestimated their weight had higher baseline BMI z-scores and an increased relative risk (RR) of overweight/obesity (RR 1.51, 95% CI 1.15–1.99). Children who misperceived themselves as underweight or overweight had stronger weight-change intention than their counterparts. However, children with weight-change intention did not develop greater changes in dietary intakes, physical activities, or sedentary behaviors than those without intention. There was no significant association between weight-change intention and incidence after adjusting for BMI z-scores at baseline. Self-reported improving diet, increasing physical activity, and dieting were associated with certain actual behavioral improvements and smaller increase in BMI z-score, but not associated with decreased risk of overweight/obesity.

**Conclusion:**

The 9-months cumulative incidence of overweight/obesity was 2.77% among Chinese children. Weight-change intention was not associated with incidence of overweight/obesity in normal-weight children, regardless of their weight perception. More importantly, children’s weight-change intention was insufficient in achieving desirable behavioral changes. Future overweight and obesity prevention programs should directly target on promoting children’s actual behavioral changes.

**Electronic supplementary material:**

The online version of this article (10.1186/s12966-018-0737-6) contains supplementary material, which is available to authorized users.

## Introduction

The substantial increase in childhood overweight and obesity poses a major public health problem worldwide [1, 2]. Fueled by the new dietary patterns and sedentary lifestyles, childhood obesity in China also increased rapidly during the past two decades [3]. In 2013, approximately 23.0% and 14.0% of Chinese boys and girls aged 2–19 years were overweight or obese [2]. As childhood obesity damages health beginning early in life, it is imperative to take early intervention. Identification of promising intervention strategies requires a clear understanding of modifiable factors contributing to childhood obesity. Specifically, gaps persist in our knowledge about how psychosocial factors impact the development of obesity among children [4].

Weight-change intention is an important psychosocial factor for effective interventions on childhood obesity. It is supposed to mediate the relationship between weight perception and weight-related behaviors, subsequently lead to weight change [5–10]. Generally, children with overweight perception showed stronger intention to lose weight and had higher reporting rates of weight-control behaviors, such as dieting and exercise [5, 6, 8]. Inconsistent to these findings, several prospective studies found that overweight perception was related to larger weight gain and increased risk of overweight and obesity among normal-weight adolescents [11–13]. A suggested explanation for the increased risk was that these adolescents may be more likely to adopt unhealthy weight-control behaviors, resulting in subsequently overweight and obesity [12, 13]. However, children’s actual changes in dietary intakes, physical activities and sedentary behaviors were not empirically examined in these studies.

Several additional gaps remain in the existing literature. First, the prevalence of overweight/obesity refers to the proportion of children who are overweight/obesity at a specific period of time, while the incidence of overweight/obesity reflects the frequency of children who become overweight/obesity during a particular time period [14]. Although the prevalence of overweight/obesity in Chinese children has been well investigated, little is known about the incidence [3]. Examining the incidence may provide insights into the development of obesity and inform intervention [15, 16]. Second, according to the Theory of Planned Behavior, weight related intention (e.g., lose weight) is an important first step for proceeding to behaviors in adults [17–19]; nevertheless, the practical effect of weight-change intention on behavioral changes among normal-weight children remained unclear. What’s more, whether weight-change intention was related to a decreased risk of overweight/obesity was seldom investigated.

Thus, we followed up a national sample of Chinese children aged 6–17 years for 9 months to assess whether weight-change intention showed effects on childhood weight status and weight-related behaviors. We were particularly interested in examining the following: (1) national incidence of childhood overweight/obesity among Chinese children; (2) the association between weight-change intention and the incidence of overweight/obesity; and (3) whether changes in dietary intakes, physical activities and sedentary behaviors differed by weight-change intention.

## Methods

### Study design and participants

Data were obtained from a national multi-center random controlled school-based obesity intervention program conducted from September, 2013 to June, 2014 in China (Registration number: NCT02343588). Using multistage cluster sampling method, 94 primary and secondary schools from 7 provinces were selected and randomized into control or intervention group. Study design and sampling procedures were reported in detail elsewhere [20]. In the current study, we only included initial normal-weight (*n* = 20,141) or underweight (*n* = 1980) children from the control group. These children were all enrolled in baseline measurement and 98.5% of them (*n* = 21,796, 19,887 and 1909 of them were normal- and underweight respectively) participated the follow-up data collection. The analytic sample consists of the 21,796 children with baseline and follow-up data. All children and their parents signed informed consents and the protocol was approved by the Ethical Committee of the Peking University.

### Anthropometric measurement

#### BMI, BMI z-scores and weight status

Weight and height were objectively measured both at baseline and follow-up. Children were required to remove their shoes and socks. Afterwards, their height and weight were measured by qualified technicians in increments of 0.1 cm and 0.1 kg, respectively, while they were wearing light clothing. Body mass index (BMI) was calculated (weight (kg)/height (m^2^)). Sex- and age- specific BMI z-scores were computed using the reference provided by the World Health Organization (WHO) [21]. Weight status was classified as underweight, normal weight, overweight and obesity using the WHO growth reference curves for 6-year-old children, and Chinese criteria [22] for children aged 7–18 years.

#### Onset of overweight and obesity and incidence

Binary indicators of incident overweight and obesity were constructed. Change from underweight or normal weight at baseline to overweight (including obesity) at follow-up was coded as incident overweight (obesity). All the participants in this study were normal- or underweight at baseline and thus were at risk for incident overweight and obesity. Cumulative incidence was calculated by dividing the number of newly overweight or obese children by the number of children at risk (*n* = 21,796). While incidence rate was obtained by dividing the number of new overweight or obesity cases by the number of person-years of follow-up, which was expressed as a rate per 1000 person-years.

### Questionnaire survey

The methods used for data collection have been described elsewhere [10]. In brief, well-trained investigators distributed child-reported and parent-reported questionnaires to children in class and interpreted all of the items in child-reported questionnaires in detail. Children were instructed to deliver the questionnaires to their parents. Parents were instructed to fill out the parent-reported questionnaires, assist their children in completing the child-reported questionnaires and return the questionnaires in 3 days. All questionnaires were checked for logicality and integrity upon return. Investigators were trained to verify the illogical answers and obtain answers for the missing items by asking the children or calling their parents.

#### Demographics information

Parents’ educational level, monthly family income and family location were based on parent report at baseline.

#### Weight perception

Weight perception was assessed at baseline by comparing their perceived and actual weight status. Perceived weight was collected by asking children ‘How would you describe your weight?’ with response options including ‘very thin’, ‘slightly thin’, ‘about right’, ‘slightly fat’ and ‘very fat’. If ‘about right’ were selected, the normal-weight children were considered as having accurate weight perception; if not, they were considered as having weight misperception, which can be underestimation or overestimation.

#### Weight-change intention

Children’s weight-change intention was captured at baseline by asking them ‘Do you want to change your weight status?’ with response options including ‘no’, ‘rather no’, ‘not sure’, ‘rather yes’, and ‘yes’. The 5-point responses were reclassified as trinary variables (no/not sure/yes).

#### Weight-control practices

Weight control practices were binary coded at follow-up. Children reported whether they had used one or more of the following 4 practices to lose weight (yes/no) during the last 3 months: improving diet (e.g. eat more fruit and vegetable, eat less high-energy snacks), increasing physical activity, dieting, or taking weight loss drugs.

#### Dietary intakes, physical activities and sedentary behaviors

Dietary intakes were assessed using 8 items. Children reported the frequency (days) and amount (servings) of fruits, vegetables, meat products and sugar-sweetened beverages intakes during the last 7 days. The average daily consumptions of these food intakes were calculated. Children reported amount (servings) of milk products, high-energy snacks, fried food, and Western fast food intake during the last 7 days. Physical activities and sedentary behaviors were assessed by 3 items respectively. Physical activities included vigorous-intensity physical activities (VPA), moderate-intensity physical activities (MPA) and walking. Children reported the frequency (days) and duration (hours and minutes in each of those days) of these activities during the last 7 days. The average daily time of physical activities were calculated. Sedentary behaviors included doing homework, viewing television and using a computer (including playing videogames). Children reported the average amount of time (hours and minutes) they spent daily on the three behaviors during the last 7 days. These variables were assessed both at baseline and follow-up. Changes were calculated as average daily consumption/time at follow-up minus that at baseline. The reliability and validity of the questionnaires of dietary intakes, physical activities and sedentary behaviors were assessed in a sample of 298 children. The results indicate that the questionnaires have acceptable reliability and validity (data not shown).

### Statistical analysis

Descriptive results were expressed as mean [standard deviation (SD)] for continuous variables and frequency for categorical variables. Differences of continuous variables between/among groups were evaluated employing *t*-test or analysis of variance. For categorical variables, chi-square tests were employed. The age-, sex- and baseline BMI z-score controlled effect of weight-change intention/self-reported weight control practices on categorical and continuous outcomes were evaluated by the Cochran–Mantel–Hansel test and general liner model respectively. Logistic regression models were run to estimate the associations between weight-change intention and incidence of childhood overweight/obesity. Sets of variables that might confound the associations were adjusted sequentially. The fully adjusted model included demographics (i.e., age, sex, ethnicity, family income, parents’ education level, location and region), children’s weight perception and BMI z-score at baseline. Analyses were conducted using SAS version 9.2 (SAS Institute, Cary, NC, USA). All tests were 2-sided and considered statistically significant at *P* < 0.05.

## Results

The baseline characteristics of the normal-weight children are represented in Table 1. Of the 19,887 children, nearly half were boys (47.1%). The mean age was 10.84 (SD 3.38) years old. Compared with girls, boys were slightly younger and had higher BMI z-scores at baseline (all *P* < 0.001). Approximately half of the fathers and mothers had an education level of junior high school or below. The majority of (44.5%) parents reported their monthly family income were in the range of 2000–12,000 RMB.

**Table 1:** General characteristics of children with normal-weight at baseline by sex

	Overall	Boys	Girls	*P* ^a^
	19,887	9361 (47.1%)	10,526 (52.9%)	
Age (years) (mean ± SD)^*^	10.84 ± 3.38	10.74 ± 3.36	10.93 ± 3.39	< 0.001
6–11.9	11,242 (56.5%)	5481 (58.6%)	5761 (54.7%)	
12–18	8645 (43.5%)	3880 (41.5%)	4765 (45.3%)	
Ethnicity				0.442
Han	16,618 (94.3%)	7755 (94.2%)	8863 (94.4%)	
Others	1006 (5.7%)	482 (5.9%)	524 (5.6%)	
Paternal education level				< 0.001
Junior high school or below	7855 (47.7%)	3748 (49.2%)	4107 (46.3%)	
Senior high school	4505 (27.4%)	2065 (27.1%)	2440 (27.5%)	
Junior college	2127 (12.9%)	939 (12.3%)	1188 (13.4%)	
College or above	1987 (12.1%)	860 (11.3%)	1127 (12.7%)	
Maternal education level				< 0.001
Junior high school or below	8529 (51.9%)	4061 (53.6%)	4468 (50.5%)	
Senior high school	4107 (25.0%)	1849 (24.4%)	2258 (25.5%)	
Junior college	2049 (12.5%)	875 (11.6%)	1174 (13.3%)	
College or above	1741 (10.6%)	786 (10.4%)	955 (10.8%)	
Monthly family income				< 0.001
< 2000 RMB	1573 (7.9%)	702 (7.5%)	871 (8.3%)	
2000–5000 RMB	4293 (21.6%)	1985 (21.2%)	2308 (21.9%)	
5000–12,000 RMB	4560 (22.9%)	2089 (22.3%)	2471 (23.5%)	
≥ 12,000 RMB	1487 (7.5%)	690 (7.4%)	797 (7.6%)	
Refuse to answer	7974 (40.1%)	3895 (41.6%)	4079 (38.8%)	
Location				0.410
Urban	13,111 (65.9%)	6144 (65.6%)	6967 (66.2%)	
Rural	6776 (34.1%)	3217 (34.4%)	3559 (33.8%)	
Region				0.171
Liaoning	2870 (14.4%)	1326 (14.2%)	1544 (14.7%)	
Tianjin	2819 (14.2%)	1368 (14.6%)	1451 (13.8%)	
Shanghai	2849 (14.3%)	1316 (14.1%)	1533 (14.6%)	
Ningxia	1993 (10.0%)	934 (10.0%)	1059 (10.1%)	
Chongqing	3389 (17.0%)	1616 (17.3%)	1773 (16.8%)	
Hunan	3040 (15.3%)	1467 (15.7%)	1573 (14.9%)	
Guangdong	2927 (14.7%)	1334 (14.3%)	1593 (15.1%)	
BMI z-score at baseline^b^ (mean ± SD)^*^	−0.17 ± 0.70	−0.11 ± 0.71	−0.22 ± 0.69	< 0.001
< 0	11,442 (57.5%)	5142 (54.9%)	6300 (59.9%)	
≥ 0.00~	2301 (11.6%)	1051 (11.2%)	1250 (11.9%)	
0.25~	2113 (10.6%)	996 (10.6%)	1117 (10.6%)	
0.50~	1838 (9.2%)	886 (9.5%)	952 (9.0%)	
≥ 0.75	2193 (11.0%)	1286 (13.7%)	907 (8.6%)	

^a^Difference between boys and girls; ^b^ BMI z-score was calculated based on the WHO standard; ^*^P < 0.001. Abbreviations: *RMB* Renminbi, *BMI* body mass index, *SD* standard deviation

**Fig. 1:**
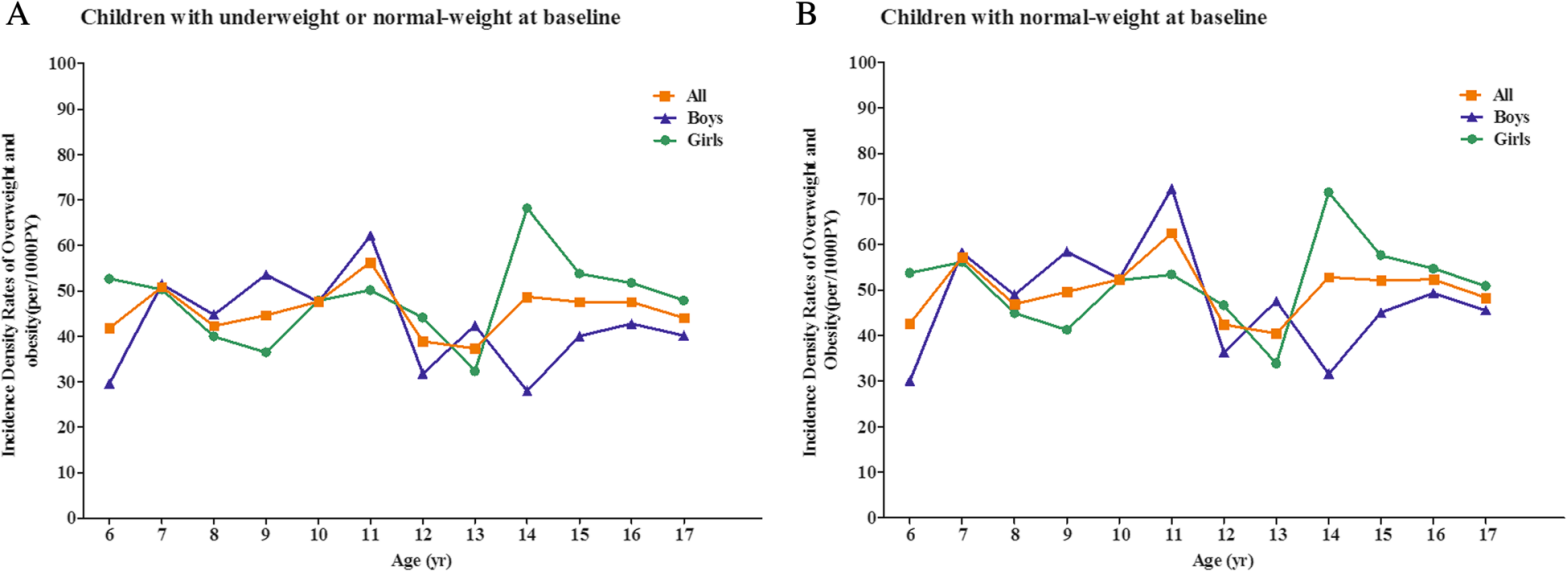
Incidence density rates of overweight/obesity among children by sex and age. Shown are the incidence density rates according to sex among children with under- or normal-weight (**a**) and with normal-weight only (**b**) at baseline

In addition to the overall cumulative incidence and incidence rate of overweight/obesity, we also estimated the age-specific rates of overweight/obesity for age group. As shown Additional file 1, approximately 3.03% (2.98% for boys and 3.08% for girls) of normal-weight children became overweight or obese during the nine-month follow-up, with the age-specific cumulative incidence ranging from 2.47 to 3.78%. An overall incidence rate was 49.23 per 1000 person-years (PY) among them (Fig. 1b). When under-weight children were also included, the cumulative incidence was 2.77% (2.68% for boys and 2.85% for girls), ranging from 2.28 to 3.39% by age (Additional file 1), and the incidence rate was 44.96 per 1000PY (Fig. 1a).

As listed in Table 2, no sex difference in cumulative incidence of overweight/obesity was observed. The highest incidence of overweight/obesity in boys according to maternal educational levels was among those whose mothers graduated from College or above (*P* < 0.01), while the association was not observed among girls. Higher incidences were observed particularly in Liaoning, Tianjin and Shanghai (*P* < 0.001). Among children with a BMI z-score ≥ 0.75 at baseline, 17.78% of them (15.16% in boys and 21.5% in girls) became overweight/obesity at follow up, whereas only 0.31% were observed among those who had a baseline BMI z-score below zero.

**Table 2:** Cumulative incidence of overweight/obesity among children with normal-weight by sex

	All		Boys		Girls	
	% (95% CI)	*P*	% (95% CI)	*P*	% (95% CI)	*P*
	3.03 (2.79, 3.27)		2.98 (2.64, 3.33)		3.08 (2.75, 3.41)	
Age(years)		0.689		0.234		0.706
6–11.9	3.09 (2.77, 3.41)		3.16 (2.69, 3.62)		3.02 (2.58, 3.46)	
12–17	2.96 (2.60, 3.32)		2.73 (2.22, 3.25)		3.15 (2.65, 3.64)	
Ethnicity		0.644		0.558		0.937
Han	2.94 (2.68, 3.19)		2.95 (2.58, 3.33)		2.92 (2.57, 3.27)	
Others	2.68 (1.69, 3.68)		2.49 (1.10, 3.88)		2.86 (1.43, 4.29)	
Paternal education level		0.650		0.549		0.109
Junior high school or below	3.04 (2.66, 3.42)		2.80 (2.27, 3.33)		3.26 (2.72, 3.81)	
Senior high school	2.77 (2.30, 3.25)		3.15 (2.39, 3.90)		2.46 (1.84, 3.07)	
Junior college	3.10 (2.37, 3.84)		2.77 (1.72, 3.82)		3.37 (2.34, 4.39)	
College or above	2.62 (1.92, 3.32)		3.26 (2.07, 4.44)		2.13 (1.29, 2.97)	
Maternal education level		0.880		0.008		0.099
Junior high school or below	2.86 (2.51, 3.21)		2.54 (2.05, 3.02)		3.16 (2.64, 3.67)	
Senior high school	3.09 (2.56, 3.62)		3.14 (2.34, 3.93)		3.06 (2.35, 3.77)	
Junior college	3.03 (2.28, 3.77)		3.31 (2.13, 4.50)		2.81 (1.87, 3.76)	
College or above	2.81 (2.04, 3.59)		4.20 (2.80, 5.60)		1.68 (0.86, 2.49)	
Monthly family income		0.083		0.067		0.315
< 2000 RMB	2.35 (1.60, 3.10)		1.85 (0.85, 2.85)		2.76 (1.67, 3.84)	
2000–5000 RMB	3.10 (2.58, 3.62)		3.58 (2.76, 4.39)		2.69 (2.03, 3.35)	
5000–12,000 RMB	3.46 (2.93, 4.00)		3.40 (2.62, 4.18)		3.52 (2.79, 4.25)	
≥ 12,000 RMB	2.29 (1.53, 3.05)		2.17 (1.09, 3.26)		2.38 (1.32, 3.44)	
Refuse to answer	3.02 (2.65, 3.40)		2.80 (2.28, 3.32)		3.24 (2.69, 3.78)	
Location		0.573		0.810		0.587
Urban	3.08 (2.79, 3.38)		3.01 (2.58, 3.44)		3.14 (2.73, 3.55)	
Rural	2.94 (2.53, 3.34)		2.92 (2.34, 3.50)		2.95 (2.39, 3.51)	
Region		< 0.001		< 0.001		0.244
Liaoning	4.29 (3.54, 5.03)		4.98 (3.81, 6.15)		3.69 (2.75, 4.63)	
Tianjin	3.19 (2.54, 3.84)		2.70 (1.84, 3.56)		3.65 (2.69, 4.62)	
Shanghai	3.40 (2.74, 4.07)		3.88 (2.83, 4.92)		3.00 (2.15, 3.85)	
Ningxia	2.81 (2.08, 3.54)		2.46 (1.47, 3.46)		3.12 (2.07, 4.16)	
Chongqing	2.74 (2.19, 3.29)		2.23 (1.51, 2.95)		3.21 (2.39, 4.04)	
Hunan	2.20 (1.68, 2.73)		1.77 (1.10, 2.45)		2.61 (1.82, 3.39)	
Guangdong	2.63 (2.05, 3.21)		3.00 (2.08, 3.91)		2.32 (1.58, 3.06)	
BMI z-score at baseline^a^ (mean (95CI%))		< 0.001		< 0.001		< 0.001
< 0	0.31 (0.20, 0.41)		0.23 (0.10, 0.37)		0.37 (0.22, 0.51)	
> 0.00~	0.57 (0.26, 0.87)		0.67 (0.17, 1.16)		0.48 (0.10, 0.86)	
0.25~	2.18 (1.55, 2.80)		1.81 (0.98, 2.63)		2.51 (1.59, 3.42)	
0.50~	6.47 (5.35, 7.60)		5.30 (3.83, 6.78)		7.56 (5.88, 9.24)	
≥ 0.75	17.78 (16.18, 9.38)		15.16 (13.2, 17.12)		21.5 (18.83, 24.17)	

^a^BMI z-score was calculated based on the WHO standard

Abbreviations: *CI* confidence interval, *RMB* Renminbi, *BMI* body mass index

Overall, 44.40% of children intended to change weight status at baseline, while the corresponding proportion was much lower in children with accurate weight perception (31.5%) than that in underestimation (50.1%) or overestimation (83.1%) group (Table 3). When compared to children with accurate weight perception, though children with overweight perception had smaller increase in BMI z-score (*P* < 0.001), they had higher risk of overweight/obesity after adjusted covariates [relative risk (RR) =1.51, 95% confidence interval (CI): 1.15, 1.99)]. Surprisingly, children who intended to change weight status showed greater increase in BMI z-score at follow-up than those who did not (*P* < 0.01). Children with weight-change intention had higher risk for overweight/obesity (RR = 1.67, 95% CI: 1.08, 2.59) only in those with accurate weight perception. However, the association became insignificant after adjusting for BMI z-score at baseline and other covariates (Table 3).

**Table 3:** Cumulative incidence of overweight/obesity, and its association with weight-related attitudes

Weight-related attitudes	%	BMI z-score ^*^ at baseline	Changes in BMI z-score^*^	Cumulative Incidence	RR (95% CI)		
				% (95% CI)	Model 1 ^d^	Model 2 ^e^	Model 3 ^f^
All children							
Self-weight perception							
Underestimation	34.28	−0.59 ± 0.61 ^b^	0.12 ± 0.41 ^c^	0.65 (0.45, 0.86)	0.19 (0.13, 0.27)	0.88 (0.61, 1.28)	0.71 (0.45, 1.12)
Accurate estimation	52.98	− 0.01 ± 0.64	0.11 ± 0.39	3.01 (2.66, 3.35)	1.00	1.00	1.00
Overestimation	12.74	0.35 ± 0.56	0.07 ± 0.36	8.67 (7.50, 9.84)	**3.19 (2.55, 3.98)**	**1.49 (1.18, 1.89)**	**1.51 (1.15, 1.99)**
Willingness to change weight status							
Yes	44.40	−0.15 ± 0.73 ^b^	0.11 ± 0.40	3.65 (3.24, 4.07)	**1.59 (1.12, 2.26)**	1.16 (0.80, 1.68)	1.14 (0.78, 1.66)
Not sure	11.00	−0.14 ± 0.68	0.11 ± 0.39	2.03 (1.40, 2.66)	1.00	1.00	1.00
No	44.50	−0.18 ± 0.68	0.09 ± 0.39	2.42 (2.08, 2.76)	1.25 (0.88, 1.77)	1.36 (0.94, 1.97)	1.35 (0.93, 1.96)
Underestimation							
Willingness to change weight status ^a^							
Yes	50.10	−0.64 ± 0.60 ^b^	0.10 ± 0.40 ^c^	0.50 (0.25, 0.76)	0.65 (0.24, 1.81)	0.74 (0.26, 2.13)	0.77 (0.25, 2.35)
Not sure	10.80	−0.56 ± 0.61	0.13 ± 0.41	0.78 (0.10, 1.46)	1.00	1.00	1.00
No	39.10	−0.55 ± 0.61	0.13 ± 0.42	0.81 (0.45, 1.18)	1.23 (0.45, 3.32)	0.99 (0.35, 2.78)	1.07 (0.36, 3.18)
Accurate estimation							
Willingness to change weight status							
Yes	31.50	0.03 ± 0.63 ^b^	0.12 ± 0.41 ^c^	3.34 (2.69, 4.00)	**1.67 (1.08, 2.59)**	1.16 (0.73, 1.84)	1.13 (0.71, 1.81)
Not sure	12.40	0.04 ± 0.61	0.11 ± 0.39	2.37 (1.49, 3.25)	1.00	1.00	1.00
No	56.10	−0.04 ± 0.65	0.08 ± 0.37	2.96 (2.50, 3.42)	1.24 (0.82, 1.88)	1.40 (0.90, 2.16)	1.38 (0.89, 2.15)
Overestimation							
Willingness to change weight status							
Yes	83.10	0.36 ± 0.55 ^b^	0.10 ± 0.37	9.18 (7.86, 10.50)	1.94 (0.89, 4.24)	1.34 (0.59, 3.07)	1.27 (0.54, 2.95)
Not sure	6.00	0.34 ± 0.57	0.08 ± 0.34	5.26 (1.47, 9.06)	1.00	1.00	1.00
No	10.90	0.29 ± 0.57	0.07 ± 0.35	6.64 (3.49, 9.78)	1.27 (0.51, 3.18)	1.29 (0.49, 3.43)	1.23 (0.45, 3.34)

Numbers in bold indicate statistically significant associations

^*^BMI z-score was calculated based on the WHO standard; ^a^ Significant difference of weight-change intentions among children with different weight perceptions, *P* < 0.001; ^b^ Significant difference among children with different weight-perception or weight-change intentions after adjusting for age and sex, *P* < 0.001; ^c^ Significant difference among children with different weight perceptions or weight-change intentions after adjusting for age, sex and BMI z-score at baseline, *P* < 0.001; ^d^ Adjusted for age, sex and self-perception of weight status (all children only); ^e^ Adjusted for age, sex, BMI z-score and self-perception of weight status (all children only); ^f^ Adjusted for age, sex, BMI z-score, income, paternal education level, maternal education level, ethnicity, location and region and self-perception of weight status (all children only)

Abbreviations: *BMI* body mass index, *CI* confidence interval, *RR* relative risk

The actual changes in dietary intakes (Additional file 2), physical activities and sedentary behaviors (Additional file 3) were largely similar between different intention groups. Children with accurate weight perception and intention to change weight had more decrease in intakes of fried food than those who did not (*P* = 0.02). Among children with overweight perception, those who intended to change weight had greater increase in intakes of fruit (*P* = 0.02) than those who did not.

Table 4 shows that 59.43% of the children adopted at least one of the four practices to lose weight during the last three months before follow-up. The corresponding proportion was also up to over 50% in those without weight change intention. Children were more likely to adopt improving diet (45.11%) and increasing physical activity (49.91%) than dieting (7.19%) and taking weight loss drugs (2.28%).

**Table 4:** Self-reported weight control practices among children by weight-related attitudes

Weight-related attitudes	Weight-control practice ^a^	improving diet	increasing physical activity	dieting	taking weight loss drugs
	% (95% CI)	% (95% CI)	% (95% CI)	% (95% CI)	% (95% CI)
All children	59.43 (58.71, 60.17)	45.11 (44.37, 45.85)	49.91 (49.17, 50.65)	7.19 (6.81, 7.57)	2.28 (2.06, 2.50)
Willingness to change weight status					
Yes	**65.73 (64.61, 66.85)**	**50.59 (49.41, 51.76)**	**54.40 (53.23, 55.57)**	**9.39 (8.70, 10.08)**	2.28 (1.93, 2.63)
Not sure	**58.71 (56.39, 61.04)**	**43.80 (41.47, 46.14)**	**49.88 (47.53, 52.24)**	**6.73 (5.55, 7.91)**	2.61 (1.86, 3.36)
No	**53.58 (52.41, 54.75)**	**39.85 (38.70, 41.00)**	**46.11 (44.94, 47.28)**	**4.69 (4.19, 5.18)**	2.04 (1.71, 2.37)
*P* ^b^	**< 0.001**	**< 0.001**	**< 0.001**	**< 0.001**	0.277
Underestimate	49.93 (48.58, 51.27)	36.17 (34.89, 37.46)	43.20 (41.88, 44.53)	5.09 (4.50, 5.67)	1.98 (1.61, 2.36)
Willingness to change weight status					
Yes	49.70 (47.79, 51.60)	35.99 (34.18, 37.81)	43.13 (41.26, 45.01)	4.96 (4.14, 5.79)	1.92 (1.40, 2.44)
Not sure	50.61 (46.51, 54.72)	38.50 (34.52, 42.48)	42.51 (38.46, 46.55)	7.52 (5.36, 9.68)	2.80 (1.45, 4.16)
No	50.00 (47.86, 52.14)	35.83 (33.78, 37.88)	43.47 (41.35, 45.58)	4.53 (3.64, 5.42)	1.86 (1.28, 2.43)
*P* ^b^	0.499	0.447	0.599	0.513	0.894
Accurate estimate	61.43 (60.38, 62.48)	46.86 (45.79, 47.93)	52.22 (51.15, 53.29)	6.46 (5.93, 6.99)	2.19 (1.88, 2.51)
Willingness to change weight status					
Yes	**72.54 (70.84, 74.25)**	**56.71 (54.83, 58.60)**	**60.60 (58.74, 62.46)**	**9.88 (8.74, 11.02)**	2.24 (1.68, 2.81)
Not sure	**62.45 (59.48, 65.42)**	**46.84 (43.79, 49.89)**	**53.40 (50.35, 56.45)**	**6.08 (4.61, 7.55)**	2.74 (1.74, 3.74)
No	**54.84 (53.41, 56.28)**	**41.21 (39.80, 42.62)**	**47.17 (45.74, 48.60)**	**4.58 (3.98, 5.18)**	2.03 (1.62, 2.44)
*P* ^b^	**< 0.001**	**< 0.001**	**< 0.001**	**< 0.001**	0.209
Overestimate	77.81 (75.98, 79.64)	61.56 (59.43, 63.69)	60.83 (58.69, 62.98)	14.38 (12.84, 15.92)	2.88 (2.14, 3.62)
Willingness to change weight status					
Yes	**80.86 (78.95, 82.78)**	**64.60 (62.28, 66.91)**	**62.71 (60.36, 65.05)**	**15.77 (14.00, 17.54)**	2.89 (2.07, 3.70)
Not sure	**65.08 (56.75, 73.41)**	**42.52 (33.91, 51.13)**	**55.91 (47.26, 64.55)**	**7.87 (3.19, 12.56)**	0.79 (0.00, 2.34)
No	**61.68 (55.16, 68.20)**	**49.54 (42.90, 56.18)**	**48.85 (42.19, 55.50)**	**8.29 (4.62, 11.97)**	4.19 (1.51, 6.87)
*P* ^b^	**< 0.001**	**< 0.001**	**< 0.001**	**0.023**	0.596

Numbers in bold indicate statistically significant differences with *P* values

^a^ Self-reported at least one of the four weight control practices; ^b^ Adjusted for age and sex; similar result were obtained when additional adjusted for BMI z-score at baseline

As shown in Table 5, after adjusting for age, sex and baseline BMI z-score, children who self-reported improving diet, increasing PA, or dieting had smaller increase in BMI z-score (*P* < 0.05). However, their new-onset overweight/obesity was not significantly lower than their counterparts. Indeed, children reported improving dietary had greater increase in intakes of fruit and vegetables, and greater decrease in sugar-sweetened beverages and high-energy snacks consumption when compared with those who did not (Additional file 4). Similarly, children reported increasing physical activity had greater increase in MPA, VPA, MVPA and walking than those who did not (Additional file 5).

**Table 5:** Changes in BMI z-score and cumulative incidence of overweight/obesity among children by self-reported weight control practices

Self-reported weight control practice in past three months	Changes in BMI z-score^*^				Cumulative incidence	RR (95% CI)	
	mean ± SD	*P*	*P* ^b^	*P* ^c^	% (95% CI)	Model 1 ^b^	Model 2 ^c^
Weight-control practice ^a^		**< 0.001**	**0.015**	**< 0.001**			
Yes	0.09 ± 0.40				3.77 (3.40, 4.14)	**2.14 (1.75, 2.63)**	1.03 (0.83, 1.29)
No	0.11 ± 0.41				1.80 (1.49, 2.11)	1.00	1.00
Improving diet		**0.002**	0.317	**< 0.001**			
Yes	0.09 ± 0.39				3.83 (3.40, 4.25)	**1.68 (1.41, 2.01)**	0.98 (0.81, 1.19)
No	0.10 ± 0.41				2.31 (2.01, 2.61)	1.00	1.00
Increasing physical activity		**0.015**	**0.018**	**< 0.001**			
Yes	0.09 ± 0.39				3.76 (3.36, 4.16)	**1.72 (1.44, 2.06)**	1.00 (0.83, 1.22)
No	0.10 ± 0.41				2.22 (1.91, 2.53)	1.00	1.00
Dieting		**< 0.001**	0.240	**0.028**			
Yes	0.06 ± 0.39				3.99 (2.91, 5.08)	**1.39 (1.03, 1.87)**	1.16 (0.84, 1.61)
No	0.10 ± 0.40				2.89 (2.63, 3.15)	1.00	1.00
Taking weight loss drugs		0.547	0.606	0.657			
Yes	0.11 ± 0.40				2.77 (1.16, 4.39)	0.93 (0.51, 1.70)	0.95 (0.50, 1.82)
No	0.10 ± 0.40				2.98 (2.72, 3.23)	1.00	1.00

Numbers in bold indicate statistically significant differences or associations

^*^BMI z-score was calculated based on the WHO standard; ^a^ Self-reported at least one of the four weight control practices; ^b^ Adjusted for age and sex; ^c^ Adjusted for age, sex and BMI z-score at baseline

Abbreviations: *BMI* body mass index, *SD* standard deviation, *CI* confidence interval, *RR* relative risk

## Discussion

This study assessed the incidence of overweight/obesity in a national sample of Chinese children. To our knowledge, this is the first longitudinal study to explore the associations between weight-change intention and incident overweight/obesity in a national sample of children. Misperceiving oneself as underweight or overweight was related to stronger intention to change weight among normal-weight children. Children with weight-change intention were more likely to report weight-control practices as compared with their counterparts, yet they showed little actual improvements in dietary intakes, physical activities or sedentary behaviors. In addition, there were no significant association between weight-change intention and new-onset overweight/obesity. Self-reported weight-control practices were associated with certain healthy behavioral changes and less increase in weight gain, but not with incidence of overweight/obesity.

Heretofore, to our knowledge, only 1 study has reported the national incidence of childhood overweight in China [3]. About 5.1% (approximately 0.57% annually) and 14.5% (approximately 1.32% annually) of children aged 2–18 years became overweight in 1991–2000 and 2000–2011 respectively [3]. In current study, the 9-months cumulative incidence of overweight/obesity among children aged 6–18 years was 2.77%, which had shown a more than fourfold and twofold increase of the annually incidence observed during 1991–2000 and 2000–2011. This finding was consistent with the substantial increasing prevalence of childhood overweight/obesity in China [3, 23].

The incidence of overweight/obesity in boys and girls were comparable, which was seemingly inconsistent with the sex difference in prevalence of overweight/obesity [23, 24]. This inconsistency may result from the fact that sex difference in prevalence is already evident at preschool age [16, 25]. Our data also support this suggestion that 30.4% of 6-year-old boys while 16.7% of 6-year-old girls were overweight/obese at baseline. The incidence of overweight/obesity was constant across ages. Similar results were obtained from studies in United States showing that the majority of childhood obesity occurred during the preschool years and the incidence declined with age [16, 26].

Like previous studies [13, 27], our results demonstrated that initial body weight was a strong predictor of becoming overweight/obesity. Approximately 65% of the new cases occurred among children with a BMI z-score ≥ 0.75 at baseline (data not shown). Similarly, about 75% of American children who became obese between the ages of 5 and 14 years had a BMI z-score above 0.52 at baseline [16]. These findings indicate that overweight and obesity intervention needs to expand the target population to children who are in the range of normal BMI, especially those who are most susceptible to becoming overweight or obesity in less than a year.

Normal-weight children with overweight perception had increased risk of overweight/obesity. This finding is consistent with prior findings [12, 13, 28]. However, we should note that these children actually had less weight gain than their counterparts. They had stronger intention to change weight and were more likely to adopt weight-control practices than those with accurate weight perception, which was in line with previous findings among adolescents and adults [8, 29–32]. However, these children also had a much higher initial BMI z-score. These findings suggest that the increased risk of overweight/obesity in children with overweight perception result from the fact that even a smaller weight gain can make them meet the threshold of overweight/obese. Another noteworthy finding is that the majority of normal weight children adopted weight-control practices, even among those with accurate weight perception. Also, the most frequently adopted practices were both healthy (improving diet and increasing physical activities). Yet, approximately 3.01% of them failed to maintain healthy weight 9 months later. In fact, nowadays most of children have a good awareness of weight-related knowledge thanks to the public health efforts and the easy access to relevant information [10, 33, 34]. Nevertheless, prevalence of childhood overweight and obesity continues to increase [3]. These results indicate the possibility of a ‘ceiling effect’ of education about weight perception on childhood obesity prevention. Notably, a total of 2.28% children with normal weight reported taking weight loss drugs to lose weight during the last three months. The use of weight loss drugs were also reported by children without weight-change intention (2.04%). Future research is needed to explore and identify risk factors for using weight loss drugs in normal weight children, such as the product accessibility and the influence of parents, peers and the media. In addition, public efforts are needed to strengthen health education for children’s use of weight-loss drugs and to strengthen government regulation of weight-loss drugs.

Children’s weight-change intention was not significantly associated with incidence of overweight/obesity after adjusting for initial body weight, regardless of their weight perception. These findings were partly consistent with prior results among adults that weight-loss intention was not associated with actual weight loss [35]. Our data also showed that one third of children with weight-change intention did not adopt weight-control practices, while over half of the children without weight-change intention did. Thus, the insignificant associations may due to that children did not necessarily act on their intentions and that those who intended to change weight initiated the actions but failed to maintain them. Another explanation for the insignificant associations is that children’ behavioral changes may be too small to reverse the development of overweigh/obesity. Our results showed that children with weight-change intention generally had no more improvements in dietary intakes, physical activities or sedentary behaviors compared with their counterparts without an intention. Discrepancy between weight-loss intention and eating and exercise habits have been reported in youths and adults [8, 36, 37]. Our results added evidences on these studies and empirically demonstrated that there was a gap between children’s weight-change intention and actual behavioral changes.

Both individual and environmental factors could contribute to the gap. The dietary and activity patterns could be effortful to change, considering that they are regulated by intrinsic biological processes [38–40]. In particular, children have strong biological vulnerability to palatable but mostly calorie-dense foods [38, 41]. Besides, Chinese children are now exposed to the so-called “obesogenic environment”, which filled with energy-dense foods [33, 42] and emphasized driving over bicycling or walking [24, 43]. Their abilities to adopt and maintain healthy lifestyles can be undermined by current environmental factors [38, 44]. In fact, even among children with overweight perception, who showed the strongest intention to change weight status, only small behavioral improvements in fruit intake and VPA time were observed in those who intended to change weight. Accordingly, facilitating environmental changes may help reduce the amount of willpower necessary to achieve desirable lifestyles [45, 46] and should be effective for childhood obesity prevention.

Our study also provides support for the benefit of environmental/physical changes in obesity prevention. We found that weight-control practices did have favorable influence on children’s weight. Children who self-reported weight-control practices (e.g., improving diet and increasing physical activity) gained less weight than those who did not, resulting from the fact that they indeed had made slight to moderate improvement in relevant healthy behaviors. However, these children did not have decreased risk of overweight/obesity. These results revealed that though changing diet and lifestyles rely on children alone are of value for weight control, their achievable magnitude of behavioral change couldn’t reached the threshold needed to reverse the trend of new onset of overweight/obesity [1].

Findings from our study point out that childhood obesity prevention should directly target on promoting children’s actual behavioral changes. Although children’s subjective weight-change intention shows some impact on their weight-control practices, they are susceptible to failures in achieving desirable healthy lifestyles and healthy weight status if we relied solely on their individual willpower [45]. Therefore, other than general promotions and interventions on children’s weight perception and weight control intention-enhancement, obesity prevention program needs to target at providing greater access to healthy food and to places for physical activity in multiple settings [47], from home to school to communities, making it easier for children to achieve desirable healthy lifestyles and healthy weight status.

Strengths of this study include the large sample size, the objectively measured weight and height and the empirical investigation of actual behavioral changes. Therefore, our study could not only reveal but also provide insight to the association between weight-change intention and the risk of overweight/obesity. This study also has several limitations. First, because the Chinese criteria are only applicable to children at age of 7–18 years and the WHO criteria are recommended for 6-year-old children in China [22, 48], we defined weight status using Chinese criteria for 7–18-year-old children and WHO criteria for 6-year-old children in this study. Using two different sets of criteria may cause potential bias on the convergence of the research results to all participants. However, similar results were obtained when the WHO criteria were applied for the whole sample, indicating the reliability of our results (data not shown). Second, data on behaviors were based on self-reported, which could have led to under- or over-reporting [49, 50]. However, this under- or over-reporting should have been consistent at baseline and at follow-up, making it unlikely to significantly influence the results. Third, the follow-up period of 9 months may not be long enough for estimating incidence of overweight/obesity accurately. However, these results are still valuable for understanding the current extent of childhood obesity epidemic in China. Further long-term longitudinal studies are warranted to monitor the trend and provide more insights to the nature of the epidemic. Finally, though we had adjusted for important demographic characteristic, we could not completely rule out residual confounding by unmeasured potential confounders.

## Conclusion

The 9-months cumulative incidence of overweight/obesity was 2.77% among Chinese children. Normal-weight children with overweight perception did not have larger weight gain but had higher risk of overweight/obesity. Children who misperceived themselves as underweight or overweight had stronger intention to change weight. However, weight-change intention was not associated with incidence of overweight/obesity among normal-weight children, regardless of their weight perception. Moreover, children’s weight-change intention was insufficient in achieving desirable behavioral changes and maintaining healthy weight status. Future overweight and obesity prevention programs should directly target on promoting children’s actual behavioral changes, providing permissive and supportive environment to facilitate them achieving healthy lifestyles.

## Additional files

Additional file 1: Cumulative incidence of overweight and obesity among children by sex and age. (DOCX 34 kb)

Additional file 2: Changes in dietary intakes among children by weight-related attitudes. (DOCX 34 kb)

Additional file 3: Changes in physical activities and sedentary behaviors among children by weight-related attitudes. (DOCX 34 kb)

Additional file 4: Self-reported weight control practice and changes in dietary intakes. (DOCX 25 kb)

Additional file 5: Self-reported weight control practice and changes in physical activities and sedentary behaviors. (DOCX 25 kb)

## Abbreviations

CI: Confidence interval
MPA: Moderate-intensity physical activities
MVPA: Moderate- or vigorous-intensity physical activities
RR: Relative risk
SD: Standard deviation
VPA: Vigorous-intensity physical activities
