# Supplementary material for: Incidence of childhood overweight and obesity and its association with weight-related attitudes and behaviors in China: a national longitudinal study
Source: Int J Behav Nutr Phys Act. 2018 Nov 3;15:108. doi: 10.1186/s12966-018-0737-6 (PMC6215687; doi:10.1186/s12966-018-0737-6)
Supplement: Supplementary file 1 — Cumulative incidence of overweight and obesity among children by sex and age. (DOCX 34 kb) [file 12966_2018_737_MOESM1_ESM.docx]

## **Additional file 1** Cumulative incidence of overweight/obesity among children by sex and age

|  | Underweight or normal weight at baseline | | | | | | | | |  | Normal weight at baseline | | | | | | | | |
| --- | --- | --- | --- | --- | --- | --- | --- | --- | --- | --- | --- | --- | --- | --- | --- | --- | --- | --- | --- |
| Age (year) | All | |  | Boys | |  | Girls | | *P ^a^* |  | All | |  | Boys | |  | Girls | | *P ^a^* |
|  | n | % (95% CI) |  | n | % (95% CI) |  | n | % (95% CI) |  |  | n | % (95% CI) |  | n | % (95% CI) |  | n | % (95% CI) |  |
|  | 21796 | 2.77 (2.55, 2.99) |  | 10430 | 2.68 (2.37, 2.99) |  | 11366 | 2.85 (2.54, 3.16) | 0.456 |  | 19887 | 3.03 (2.79, 3.27) |  | 9082 | 2.98 (2.64, 3.33) |  | 10526 | 3.08 (2.75, 3.41) | 0.689 |
| 6~ | 1841 | 2.61 (1.88, 3.34) |  | 864 | 1.85 (0.95, 2.75) |  | 977 | 3.28 (2.16, 4.39) | 0.056 |  | 1806 | 2.66 (1.92, 3.40) |  | 833 | 1.88 (0.97, 2.80) |  | 957 | 3.34 (2.20, 4.48) | 0.054 |
| 7~ | 2417 | 3.10 (2.41, 3.79) |  | 1178 | 3.14 (2.14, 4.14) |  | 1239 | 3.07 (2.11, 4.03) | 0.917 |  | 2153 | 3.48 (2.71, 4.26) |  | 1005 | 3.55 (2.43, 4.67) |  | 1111 | 3.42 (2.35, 4.49) | 0.869 |
| 8~ | 2607 | 2.57 (1.96, 3.18) |  | 1289 | 2.72 (1.83, 3.60) |  | 1318 | 2.43 (1.60, 3.26) | 0.643 |  | 2317 | 2.85 (2.17, 3.53) |  | 1111 | 2.97 (1.99, 3.95) |  | 1172 | 2.73 (1.80, 3.66) | 0.730 |
| 9~ | 2684 | 2.72 (2.10, 3.34) |  | 1286 | 3.27 (2.29, 4.24) |  | 1398 | 2.22 (1.45, 2.99) | 0.095 |  | 2412 | 3.03 (2.34, 3.71) |  | 1137 | 3.56 (2.5, 4.62) |  | 1233 | 2.51 (1.64, 3.39) | 0.133 |
| 10~ | 2137 | 2.90 (2.19, 3.61) |  | 1071 | 2.89 (1.89, 3.90) |  | 1066 | 2.91 (1.9, 3.92) | 0.985 |  | 1945 | 3.19 (2.41, 3.97) |  | 936 | 3.21 (2.10, 4.32) |  | 978 | 3.17 (2.07, 4.27) | 0.964 |
| 11~ | 678 | 3.39 (2.03, 4.76) |  | 348 | 3.74 (1.74, 5.73) |  | 330 | 3.03 (1.18, 4.88) | 0.612 |  | 609 | 3.78 (2.26, 5.29) |  | 286 | 4.35 (2.04, 6.66) |  | 310 | 3.23 (1.26, 5.19) | 0.468 |
| 12~ | 1782 | 2.41 (1.70, 3.13) |  | 755 | 1.99 (0.99, 2.98) |  | 1027 | 2.73 (1.73, 3.72) | 0.315 |  | 1637 | 2.63 (1.85, 3.40) |  | 650 | 2.26 (1.13, 3.38) |  | 972 | 2.88 (1.83, 3.93) | 0.437 |
| 13~ | 2102 | 2.28 (1.64, 2.92) |  | 1046 | 2.58 (1.62, 3.54) |  | 1056 | 1.99 (1.15, 2.83) | 0.363 |  | 1943 | 2.47 (1.78, 3.16) |  | 907 | 2.89 (1.82, 3.97) |  | 1009 | 2.08 (1.20, 2.96) | 0.251 |
| 14~ | 837 | 2.99 (1.83, 4.14) |  | 409 | 1.71 (0.45, 2.97) |  | 428 | 4.21 (2.30, 6.11) | 0.034 |  | 770 | 3.25 (1.99, 4.50) |  | 355 | 1.93 (0.51, 3.35) |  | 408 | 4.41 (2.42, 6.40) | 0.053 |
| 15~ | 1947 | 2.98 (2.22, 3.73) |  | 876 | 2.51 (1.48, 3.55) |  | 1071 | 3.36 (2.28, 4.44) | 0.272 |  | 1778 | 3.26 (2.44, 4.09) |  | 755 | 2.83 (1.66, 4.00) |  | 1001 | 3.60 (2.44, 4.75) | 0.368 |
| 16~ | 1985 | 3.02 (2.27, 3.78) |  | 915 | 2.73 (1.68, 3.79) |  | 1070 | 3.27 (2.21, 4.34) | 0.485 |  | 1808 | 3.32 (2.49, 4.14) |  | 770 | 3.14 (1.93, 4.36) |  | 1013 | 3.46 (2.33, 4.58) | 0.715 |
| 17~ | 779 | 2.82 (1.66, 3.99) |  | 393 | 2.54 (0.99, 4.10) |  | 386 | 3.11 (1.38, 4.84) | 0.635 |  | 709 | 3.10 (1.83, 4.38) |  | 337 | 2.88 (1.12, 4.64) |  | 362 | 3.31 (1.47, 5.16) | 0.740 |
| *P ^b^* |  | 0.789 |  |  | 0.582 |  |  | 0.398 |  |  |  | 0.744 |  |  | 0.845 |  |  | 0.543 |  |

^a^ Difference of incidence of overweight and obesity between boys and girls; ^b^ Difference among age groups.

Abbreviations: CI, confidence interval.
