# Supplementary material for: Incidence of childhood overweight and obesity and its association with weight-related attitudes and behaviors in China: a national longitudinal study
Source: Int J Behav Nutr Phys Act. 2018 Nov 3;15:108. doi: 10.1186/s12966-018-0737-6 (PMC6215687; doi:10.1186/s12966-018-0737-6)
Supplement: Supplementary file 2 — Changes in dietary intakes among children by weight-related attitudes. (DOCX 34 kb) [file 12966_2018_737_MOESM2_ESM.docx]

## **Additional file 2** Changes in dietary intakes among children by weight-related attitudes ^a^

| Weight-related attitudes | Changes in Food intake (serving/day) | | |  | Changes in Food intake (frequency/week) | | | | |
| --- | --- | --- | --- | --- | --- | --- | --- | --- | --- |
|  | Fruit | Vegetable | Meat |  | Milk | SSB | High-energy snacks | Fried food | Western fast food |
| **All children** | 0.04±1.33 | 0.04±1.76 | -0.04±1.42 |  | -0.02±2.92 | -0.03±0.86 | -0.05±2.36 | -0.08±1.87 | -0.11±2.63 |
| Willingness to change weight status |  |  |  |  |  |  |  |  |  |
| Yes | 0.05±1.30 | 0.01±1.74 | -0.04±1.38 |  | -0.02±2.94 | -0.03±0.88 | -0.06±2.34 | -0.12±1.94 | -0.14±2.70 |
| Not sure | 0.08±1.41 | 0.10±1.73 | -0.06±1.51 |  | 0.00±2.90 | -0.03±0.90 | -0.04±2.46 | -0.12±1.88 | -0.16±2.80 |
| No | 0.03±1.34 | 0.05±1.77 | -0.03±1.43 |  | -0.03±2.90 | -0.02±0.82 | -0.04±2.36 | -0.04±1.80 | -0.06±2.53 |
| *P* ^b^ | 0.249 | 0.352 | 0.788 |  | 0.611 | 0.990 | 0.946 | 0.115 | 0.262 |
| **Underestimate** |  |  |  |  |  |  |  |  |  |
| Willingness to change weight status |  |  |  |  |  |  |  |  |  |
| Yes | 0.07±1.29 | 0.04±1.68 | -0.01±1.40 |  | -0.18±2.92 | -0.01±0.87 | -0.05±2.32 | -0.07±1.90 | -0.07±2.64 |
| Not sure | 0.10±1.45 | 0.15±1.70 | 0.02±1.40 |  | -0.03±2.89 | -0.02±0.88 | -0.02±2.48 | -0.12±1.79 | -0.17±2.57 |
| No | 0.03±1.39 | 0.11±1.77 | -0.02±1.55 |  | -0.04±2.95 | -0.01±0.94 | -0.01±2.43 | -0.07±1.92 | -0.13±2.69 |
| *P* ^b^ | 0.440 | 0.230 | 0.871 |  | 0.252 | 0.979 | 0.806 | 0.900 | 0.603 |
| **Accurate estimate** |  |  |  |  |  |  |  |  |  |
| Willingness to change weight status |  |  |  |  |  |  |  |  |  |
| Yes | 0.01±1.31 | 0.03±1.76 | -0.05±1.31 |  | 0.06±2.92 | -0.05±0.90 | -0.06±2.33 | -0.15±1.92 | -0.16±2.79 |
| Not sure | 0.08±1.37 | 0.09±1.70 | -0.09±1.56 |  | 0.05±2.84 | -0.04±0.84 | -0.01±2.43 | -0.13±1.94 | -0.16±2.96 |
| No | 0.04±1.31 | 0.03±1.76 | -0.04±1.36 |  | -0.03±2.87 | -0.02±0.76 | -0.04±2.32 | -0.02±1.74 | -0.02±2.46 |
| *P* ^b^ | 0.467 | 0.411 | 0.628 |  | 0.768 | 0.781 | 0.767 | 0.022 | 0.147 |
| **Overestimate** |  |  |  |  |  |  |  |  |  |
| Willingness to change weight status |  |  |  |  |  |  |  |  |  |
| Yes | 0.08±1.29 | -0.04±1.81 | -0.06±1.44 |  | 0.11±2.99 | -0.04±0.87 | -0.06±2.37 | -0.13±2.03 | -0.23±2.66 |
| Not sure | 0.06±1.34 | -0.09±2.02 | -0.19±1.63 |  | -0.27±3.36 | -0.01±1.38 | -0.42±2.54 | -0.09±1.76 | -0.12±2.59 |
| No | -0.16±1.39 | 0.01±1.93 | 0.01±1.49 |  | 0.14±3.00 | -0.09±0.74 | -0.30±2.45 | -0.26±1.97 | -0.37±2.43 |
| *P* ^b^ | **0.021** | 0.843 | 0.394 |  | 0.281 | 0.515 | 0.072 | 0.530 | 0.737 |

^a^ Change = follow up – baseline; ^b^ Adjusted for age and sex; similar results were obtained when additional adjusted for BMI z-score at baseline.

Abbreviations: SSB, sugar-sweetened beverages.
