# Supplementary material for: Incidence of childhood overweight and obesity and its association with weight-related attitudes and behaviors in China: a national longitudinal study
Source: Int J Behav Nutr Phys Act. 2018 Nov 3;15:108. doi: 10.1186/s12966-018-0737-6 (PMC6215687; doi:10.1186/s12966-018-0737-6)
Supplement: Supplementary file 3 — Changes in physical activities and sedentary behaviors among children by weight-related attitudes. (DOCX 34 kb) [file 12966_2018_737_MOESM3_ESM.docx]

## **Additional file 3** Changes in physical activities and sedentary behaviors among children by weight-related attitudes ^a^

| Weight-related attitudes | Changes ^b^ in physical activity (min / day) | | | |  | Changes ^b^ in sedentary behavior (min / day) | | | |
| --- | --- | --- | --- | --- | --- | --- | --- | --- | --- |
|  | MPA | VPA | MVPA | Walking |  | Homework time | TV time | PC time | Screen time |
| **All children** | 4.75±56.84 | 4.59±52.55 | 7.35±90.99 | 5.60±89.67 |  | -1.43±78.63 | 0.37±74.78 | 4.00±78.15 | 4.09±123.52 |
| Willingness to change weight status |  |  |  |  |  |  |  |  |  |
| Yes | 4.80±53.84 | 4.09±50.20 | 6.63±85.62 | 5.62±90.85 |  | -1.47±90.85 | -0.27±78.86 | 3.98±82.36 | 3.14±131.52 |
| Not sure | 6.50±63.07 | 4.88±58.33 | 9.44±101.99 | 7.87±86.02 |  | 0.02±76.68 | 1.00±79.87 | 6.15±80.13 | 7.90±128.61 |
| No | 4.38±57.73 | 5.16±52.88 | 7.72±92.36 | 5.20±89.02 |  | -1.80±78.40 | 0.71±69.35 | 3.55±72.71 | 4.02±113.61 |
| *P* ^c^ | 0.421 | 0.866 | 0.735 | 0.476 |  | 0.638 | 0.624 | 0.575 | 0.531 |
| **Underestimate** |  |  |  |  |  |  |  |  |  |
| Willingness to change weight status |  |  |  |  |  |  |  |  |  |
| Yes | 5.67±56.01 | 6.08±50.67 | 9.21±89.02 | 5.76±94.25 |  | 2.09±94.25 | 0.00±72.83 | 6.60±79.14 | 6.21±124.42 |
| Not sure | 4.14±71.09 | 5.61±57.62 | 8.73±107.05 | 2.64±78.50 |  | -0.86±75.74 | 2.35±71.32 | 12.07±71.98 | 15.92±113.54 |
| No | 5.27±58.57 | 5.64±49.62 | 9.61±90.86 | 6.16±90.21 |  | 0.41±81.80 | -1.83±74.67 | 1.10±80.19 | -0.26±123.85 |
| *P* ^c^ | 0.883 | 0.967 | 0.973 | 0.751 |  | 0.777 | 0.460 | **0.009** | **0.037** |
| **Accurate estimate** |  |  |  |  |  |  |  |  |  |
| Willingness to change weight status |  |  |  |  |  |  |  |  |  |
| Yes | 4.50±50.88 | 2.67±52.18 | 5.15±84.84 | 6.21±87.51 |  | -1.52±87.51 | 1.14±80.48 | 2.85±80.46 | 4.17±131.08 |
| Not sure | 7.46±59.06 | 5.31±58.46 | 10.37±98.59 | 10.66±88.14 |  | 0.84±77.93 | 1.74±77.92 | 3.12±81.01 | 3.77±131.40 |
| No | 4.32±57.46 | 5.38±54.20 | 7.62±92.79 | 4.73±88.64 |  | -2.54±75.87 | 2.21±65.82 | 4.80±68.46 | 6.22±107.90 |
| *P* ^c^ | 0.297 | 0.521 | 0.559 | 0.118 |  | 0.198 | 0.757 | 0.545 | 0.697 |
| **Overestimate** |  |  |  |  |  |  |  |  |  |
| Willingness to change weight status |  |  |  |  |  |  |  |  |  |
| Yes | 3.90±54.91 | 3.02±45.87 | 4.61±81.06 | 4.58±90.51 |  | -7.38±90.51 | -2.89±86.22 | 1.48±90.69 | -3.67±143.91 |
| Not sure | 8.75±56.66 | -2.11±60.93 | 4.64±106.47 | 9.03±100.90 |  | -3.17±70.92 | -11.49±123.48 | 4.73±105.94 | 4.82±167.44 |
| No | -3.27±55.88 | -5.04±54.41 | -9.83±97.98 | 4.19±82.70 |  | -8.40±98.45 | -6.23±89.54 | 1.15±83.75 | 0.55±128.32 |
| *P* ^c^ | 0.251 | 0.076 | 0.124 | 0.868 |  | 0.868 | 0.643 | 0.944 | 0.861 |

^a^ Data are shown as mean**±**SD; ^b^ Change= follow up – baseline; ^c^ Adjusted for age and sex; similar results were obtained when additional adjusted for BMI z-score at baseline.

Abbreviations: MPA, moderate-intensity physical activities; VPA, vigorous-intensity physical activities; MVPA, moderate- or vigorous-intensity physical activities; TV, television; PC, personal computer.
