# Supplementary material for: Incidence of childhood overweight and obesity and its association with weight-related attitudes and behaviors in China: a national longitudinal study
Source: Int J Behav Nutr Phys Act. 2018 Nov 3;15:108. doi: 10.1186/s12966-018-0737-6 (PMC6215687; doi:10.1186/s12966-018-0737-6)
Supplement: Supplementary file 4 — Self-reported weight control practice and changes in dietary intakes. (DOCX 25 kb) [file 12966_2018_737_MOESM4_ESM.docx]

## **Additional file 4** Self-reported weight control practice and changes in dietary intakes ^a^

| Changes ^b^ in food intakes | Self-reported improving diet in the last three months | | | |
| --- | --- | --- | --- | --- |
|  | Yes | No | *P* ^c^ | *P* ^d^ |
| Fruit intake (serving / day) | 0.09±1.39 | 0.01±1.27 | **<0.001** | **<0.001** |
| Vegetable intake (serving / day) | 0.08±1.79 | 0.01±1.73 | **0.018** | **0.017** |
| Meat products intake (serving / day) | -0.05±1.41 | -0.03±1.43 | 0.543 | 0.576 |
| Milk intake (frequency/ week) | 0.02±2.92 | -0.04±2.91 | 0.418 | 0.453 |
| SSB intake (frequency/ week) | -0.04±0.85 | -0.01±0.86 | **0.049** | **0.046** |
| High-energy snacks intake (frequency/ week) | -0.10±2.32 | 0.00±2.38 | **0.011** | **0.011** |
| Fried food intake (frequency/ week) | -0.11±1.87 | -0.06±1.87 | 0.157 | 0.209 |
| Western fast food intake (frequency/ week) | -0.12±2.75 | -0.10±2.54 | 0.748 | 0.910 |

^a^ Data are shown as mean**±**SD. ^b^ Change = follow up – baseline. ^c^ Adjusted for age and sex; ^d^ Adjusted for age, sex and BMI z-score at baseline.

Abbreviations: SSB, sugar-sweetened beverages.
