# Supplementary material for: Incidence of childhood overweight and obesity and its association with weight-related attitudes and behaviors in China: a national longitudinal study
Source: Int J Behav Nutr Phys Act. 2018 Nov 3;15:108. doi: 10.1186/s12966-018-0737-6 (PMC6215687; doi:10.1186/s12966-018-0737-6)
Supplement: Supplementary file 5 — Self-reported weight control practice and changes in physical activities and sedentary behaviors. (DOCX 25 kb) [file 12966_2018_737_MOESM5_ESM.docx]

## **Additional file 5** Self-reported weight control practice and changes in physical activities and sedentary behaviors ^a^

| Changes ^b^ in physical activity and sedentary behaviors | Self-reported increasing physical activity in the last three months | | | |
| --- | --- | --- | --- | --- |
|  | Yes | No | *P* ^c^ | *P* ^d^ |
| **Physical activity** |  |  |  |  |
| MPA (min / day) | 7.42±60.74 | 2.08±51.93 | **<0.001** | **<0.001** |
| VPA (min / day) | 7.64±58.51 | 1.66±44.74 | **<0.001** | **<0.001** |
| MVPA (min / day) | 13.50±100.09 | 1.55±79.93 | **<0.001** | **<0.001** |
| Walking (min / day) | 8.13±93.45 | 3.70±85.35 | **0.015** | **0.019** |
| **Sedentary behavior** |  |  |  |  |
| Homework time (min / day) | -1.39±79.36 | -1.77±77.77 | 0.240 | 0.122 |
| TV time (min / day) | 0.15±76.25 | 0.55±72.94 | 0.868 | 0.951 |
| PC time (min / day) | 3.28±80.60 | 4.30±75.22 | 0.546 | 0.767 |
| Screen time (min / day) | 3.37±128.02 | 4.39±118.50 | 0.820 | 0.984 |

^a^ Data are shown as mean**±**SD. ^b^ Change = follow up – baseline; ^c^ Adjusted for age and sex; ^d^ Adjusted for age, sex and BMI z-score at baseline.

Abbreviations: MPA, moderate-intensity physical activities; VPA, vigorous-intensity physical activities; MVPA, moderate- or vigorous-intensity physical activities; TV, television; PC, personal computer.
